# Acting without Central Agent—Considerations for a Self-Model at the Cellular Level

**DOI:** 10.3389/fnhum.2017.00191

**Published:** 2017-04-19

**Authors:** Stefan Kippenberger, Johannes Kleemann, Roland Kaufmann, Markus Meissner

**Affiliations:** Department of Dermatology, Allergy and Venereology, University of Frankfurt Medical SchoolFrankfurt, Germany

**Keywords:** learning, memory, self-model, Physarum, evolution, metaphors, therapy

## Life on earth—the nervous system

Fossil discoveries suggest that life began on earth about 3.8 billion years ago. At first there were single-celled organisms with no nuclei (prokaryotes), later ~2.7 billion years ago, protozoa developed which are cells with a nucleus (eukaryotes). Only since about 800 million years have multicellular organisms (eumetazoa) emerged and with that the ability to evolve specialized cells, such as nerve cells. The function of nerve cells is to receive information from both the internal and external environment, interpret and translate it into body reactions e.g., muscle contractions which change the body's position. In radially symmetric multicellular organisms such as jellyfish, there is a loose network of nerves which condenses in higher organisms with bilateral symmetry to a central nervous system. In bilateria the main axis is mostly in the direction of movement, where, at the front, uptake of nutrients takes place. This defines the starting point for cephalization with the emergence of a cerebral ganglion. Later in evolution, according to the prosomeric model the morphology of the vertebrate forebrain emerges from segmental structures, the number, and interconnections of ganglia increased forming a brain, the place where we assume cognition and intelligence is located. The everyday neuro-fixation ignores indications that already single cells show complex behavior patterns with a kind of basal intelligence and the ability to memorize. How do single cells accomplish this? In the following we hypothesize that cells may have a kind of self-model, a representation of themselves.

## Phenomenal self-model

For planned action, the presence of a brain is a prerequisite. After a philosophical theory, for which there is increasingly reliable experimental data, the brain produces a phenomenal self-model (PSM), which creates a representation of one's body in which its upper layers are functionally anchored (Metzinger, 2003, 2008; Lenggenhager et al., 2007). The PSM creates the feeling of “mineness” and of being a self. Arbitrary and controlled acts, i.e., movements for which we possess a veto control and which are initiated by a conscious act, will be simulating within the PSM before implementation: Hence we know which motor co-ordination is necessary to grasp an object by directing the introspective attention to the process of action planning. Within a certain time frame the action can be canceled or modified. The PSM—or virtual self—enables the holder to interact with the environment in a particularly flexible and context-sensitive manner. This is especially necessary when complex environmental conditions arise that require new strategies: a dog that lacks a limb is still able to walk, but has to develop a new co-ordination pattern for the remaining limbs. This is done in the repetitive interaction between self-model and environment. This procedure has proven its versatility in the development of robots with artificial intelligence. A walking robot equipped with a self-model can compensate the shutdown of a limb by developing a new style of locomotion (Bongard et al., 2006).

## Self-model at the cellular level?

The presence of a nervous system is not a *conditio sine qua non* to receive environmental stimuli and to translate them into appropriate responses. Single-celled organisms such as amoebae or paramecia move actively and purposefully in their environment without even having a nervous system. In the following we will provide a few examples showing the impressive behavior of organisms which we call “simple.” In an experiment, a labyrinth was evenly colonized with the unicellular slime mold *Physarum polycephalum*. Then, oat flakes, the preferred food of Physarum, were placed at the entry and exit. Within a few hours Physarum retracted all branches only leaving a linear connection of the shortest distance between entry and exit (Nakagaki et al., 2000). In a similar experiment, Physarum was grown on a circular agar plate. Three oat flakes were placed at the corners of a triangle; the fungus found a link corresponding to the mathematically shortest route (Steiner's minimum tree; Nakagaki et al., 2004). In addition, Physarium can solve the complex traveling-salesman-problem by linking eight points with the shortest distances (Zhu et al., 2013). Moreover, there is evidence for learning and memory in single-celled organisms. After irritating Physarum with dry air it slows its running speed. After three irritations the cell anticipates further stimuli and slows down the running speed without drying stimulus. When irritations were permanently turned off, the memory disappeared (Saigusa et al., 2008). Also in *Paramecium caudatum*, a single-celled aquatic organism, there is evidence for learning: the cells were trained with electric shocks to discriminate the difference between light and dark (Armus et al., 2006). Tetrahymena, another ciliate, was held in minute water droplets, after release to a larger area it recapitulates the circular swimming trajectories from the confinement for a while (Kunita et al., 2016). And even in prokaryotes complex social behavior changes are observed, e.g., the switching from a planktonic to a biofilm lifestyles, a phenomenon known as quorum sensing (Ben Jacob et al., 2004; Hellingwerf, 2005).

These examples suggest a form of primitive intelligence and the presence of a self-model already at the cellular level. Since every multicellular individual organism starts as a single cell, from which during ontogenesis various cell species emerge, the question arises whether cells of our body use something like a self-model. The “large” self-model which was discussed above generates a kind of body map, which sometimes is in conflict with body sensations. An impressive example are the so-called “rubber hand” experiments where a virtual limb, a rubber hand looking like connected to the body, becomes part of the body map (Armel and Ramachandran, 2003). These experiments were proven with different experimental settings and recently extended to a whole body representation. By using a virtual reality device representing one's own body from the rear standing 2 m in front, the visual-somatosensory input becomes disrupted and the spatial unity between the self and the body becomes separated (Lenggenhager et al., 2007). A clinical example for conflict between body map and real world is the phantom limb pain which is frequently perceived after limb amputation (Ramachandran and Hirstein, 1998).

Is there a match for a self-model already at the cellular level? How does a cell recognize its extent and size? In the human body there are cells of different sizes, from the denucleated erythrocyte, the smallest cell, with 7.5 μm, to the oocyte with 250 μm and the nerve cell, with cell processes up to 1 m. Do these cells have a kind of self-model in order to calibrate their size? What happens in an experiment where one repeatedly cuts pieces from the cytoplasm? Does the cell compensate for the missing volume? In apocrine secretion (e.g., mammary gland, apocrine sweat gland) liquid-filled vesicles are extruded from the cell, which thereby loses volume. Thus, the cell becomes initially smaller. In merocrine secretion (e.g., pancreas), the cell loses volume by exocytosis, which causes an increase of cell membrane. Since in both types of secretion there is no permanent change in volume observed, cells seem to measure their size and also actively adjust to a set value—with a self-model?

## Prerequisites for a cellular self-model

**Figure 1:**
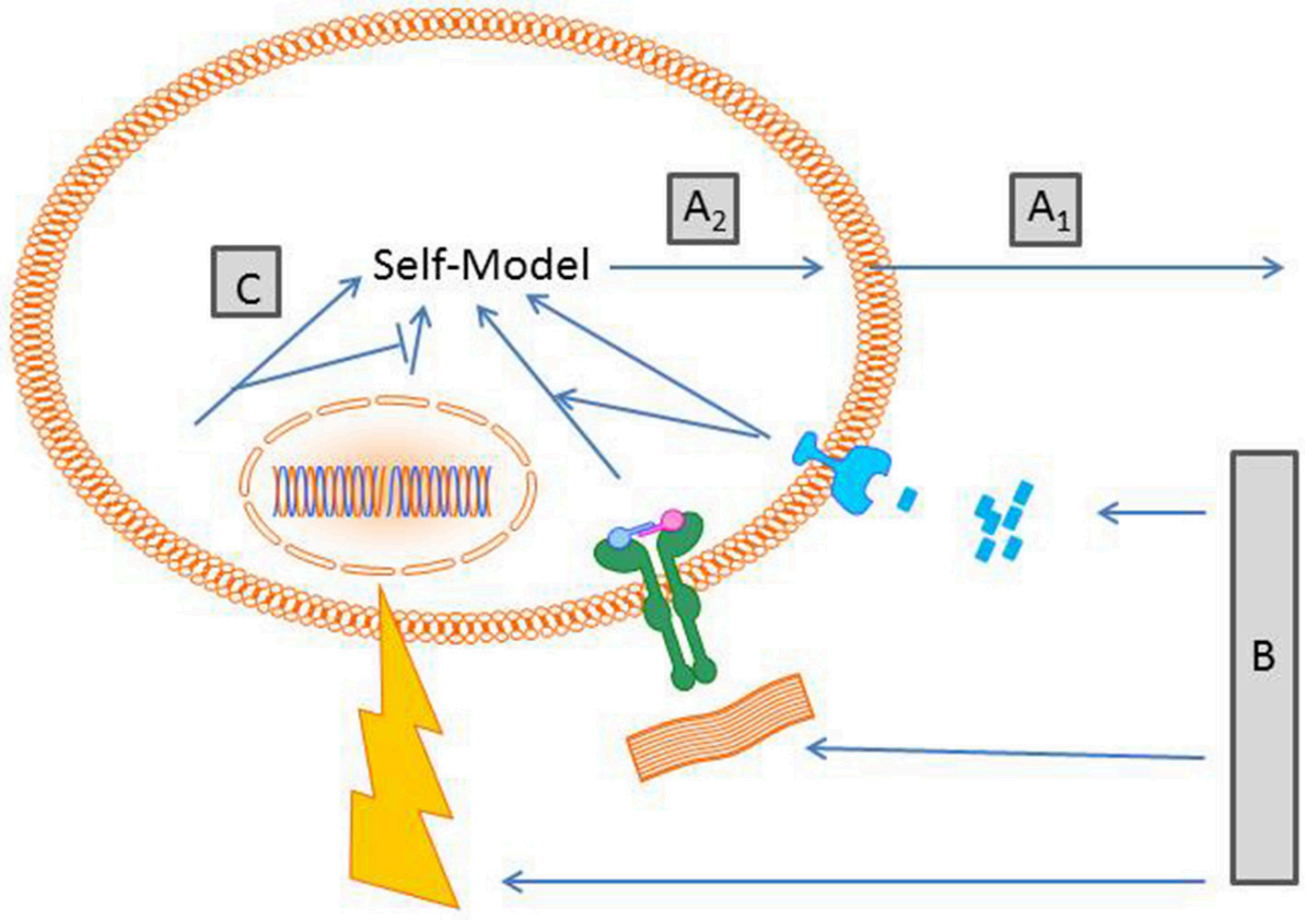
**Schematic proposal of generation of a cellular self-model**. (A_1_) Cells “act” on a probabilistic basis depending on their inherent capabilities e.g., a macrophage crawls on a substrate. Others cells that do not move “act” by expressing certain molecules/receptors. At this stage cells cast a hypothesis into the world about how the world is. (B) Cell action prompts changes of external and internal conditions. Depending on the receptor equipment cells sense different qualities of stimuli. Here exemplified are a compound gradient, receptor matrix interaction and radiation. (C) The sensory input becomes transduced in interconnected signaling cascades including elements of the cytoskeleton that generates a model of a self. Of note, the self-model is not a thing or a particular place, it is a process driven by an inherent algorithm that generates the self-model. (A_2_) The self-model generates new predictions of the world outside.

As the “large” self-model a cellular self-model makes predictions about the future taking sensory information about the present state into account. Figure 1 summarizes the components involved in the proposed concept. On the sensory level, cellular receptors can already discriminate between different information qualities, such as physical, chemical, and biological information. Here are a few examples:

- Physical stimuli: (i) temperature changes conductivity of thermosensitive TRP channels, (ii) light causes the transformation of opsin-coupled 11-cis-retinal to trans-retinal, which results in an altered membrane permeability to sodium, (iii) mechanical stretch activates cell membrane receptors of the integrin family (Kippenberger et al., 2000).
- Chemical stimuli: as a rough estimate 400 different chemical stimuli can be perceived by cells, e.g., sperm cells express olfactory receptors sensitive to bourgeonal, the dominant compound in the scent of lily of the valley. Cells are attracted by this compound and swim actively toward the odor source (Spehr et al., 2003).
- Biological stimuli: among the receptors specific for biological stimuli are the Toll-like receptors, which recognize so-called *Pathogen Associated Molecular Patterns (PAMP)*, typical structures expressed by bacteria, viruses, and fungi (Takeda and Akira, 2015).

To generate a self-model, information derived from these receptors has to be integrated. For obvious reasons, a central clearing house, such as the brain for the “large” self-model, is hard to imagine. In the proposed model, sensory information is transduced by signaling molecules forming signaling pathways which are interconnected via activating and inhibiting crosstalk. The number of players taking part in this process is immense and yet not foreseeable. Besides molecules such as kinases or phosphatases conveying the signal, there is need for molecules which organize the different signaling pathways. The assumption of cells being just sacks filled with fluid and mediators is outdated. Today we know that the cytoskeleton, consisting of actin, intermediate filament, and microtubles, is not only necessary for cell shape and cell movement but also provides an organizing lattice for proteins. Thereby, cytoskeletal proteins have impact on the probability that specific signaling molecules align in spatial proximity allowing transduction of information. For a self-model, all these components should be related in a cybernetic context. Are there individual voxels that follow an iterative algorithm creating form, similar to cellular automata or dissipative structures (Kippenberger et al., 2013, 2014)? Do cells have memristors, nonlinear variable memory elements represented e.g., by gel/sol interaction, that are triggered by internal biochemical oscillators and environmental stimuli (Pershin et al., 2009)? Important in this context is that a self-model is presumably not a thing or a state (which is hard to do) but rather an operation, a process of self-modeling. It is likely that there is no distinct physical correlate for such a self-model; maybe the self-modeling procedure is hidden in a dynamic structure, in a functional pattern of self-regulation.

## Disease and therapy—a new perspective?

The above mentioned considerations could initiate a discussion changing our view of disease and treatment. Assuming for a moment on a trial basis, that each cell has a self-model analogous to the “large” self-model (Metzinger, 2003, 2008; Lenggenhager et al., 2007). Then, a “diseased” cell, a cell that behaves no longer to the benefit of the entire organism, has a changed self-model. How can this happen? Similar to the “large” self-model, there might be a conflict between incoming information and the set values. This happens when cellular receptors fall silent (e.g., by knock-down mutation) or become permanently active (e.g., by knock-in mutation). The former situation is present in diseases such cystic fibrosis where the ion channel CFTR has lost its function (Elborn, 2016). The second situation is frequently observed in cancer, where a signaling cascade is permanently active although there is no stimulus. An example for this is the hedgehog pathway in basal cell carcinoma (Silapunt et al., 2016). The corrupted information leads to the generation of a deceptive self-model similar to phantom pain. The self-model has lost touch with reality. Through therapeutic measures, such as the pharmacological inhibition of the hedgehog pathway (by Vismodegib®), the self-model becomes corrected and former tumor cells behave again according to their original function. Typically, many tumors develop resistance to a treatment after initial therapy success. For example, melanomas with an activating BRAF mutation (V600E) respond well to inhibitors of this pathway at first (Wong and Ribas, 2016). Later many cells activate alternative signaling cascades bypassing BRAF. How can this happen? Without going into molecular details (e.g., driver mutations), it could be, as in schizophrenia, that cells project their self-model into the world without considering feedback mechanisms. If this is the case, then tumor cells “suffer from a detrimental self-model,” or strictly speaking, cells suffer from a deviant form of self-modeling, which makes inaccurate predictions. Specifically, this means that the observed behavior is the result of (a) corrupt information, or (b) a faulty cellular algorithm computing the incoming information to a harmful self-model.

The way we understand the world is largely dependent upon choosing proper metaphors that are resonant with the inner representation. This becomes particularly evident in some specific subjects such as quantum physics where we run out of speaking metaphors. The view on disease and therapy is characterized by strong metaphors which are dominated by those taken from war. Here are some examples: “to combat disease,” “immune cells patrolling,” “arsenal of drugs,” “to kill cells,” “scavenger cells,” “killer cells,” “operation,” “last line of defense,” “double hit therapy,” and many others. In the above proposed concept, disease emerges from a disturbed self-modeling process which descends to the non-neuronal cellular level. In this view a helpful therapy is the one that corrects the “rules of the game” adjusting the self-modeling algorithm. Could this change in perspective produce new solutions?

## Author contributions

SK: Discussion, investigation, writing. JK, RK, and MM: Discussion, investigation.

### Conflict of interest statement

The authors declare that the research was conducted in the absence of any commercial or financial relationships that could be construed as a potential conflict of interest.
